# Correlation of the electrophysiological profiles and sodium channel transcripts of individual rat dorsal root ganglia neurons

**DOI:** 10.3389/fncel.2014.00285

**Published:** 2014-09-19

**Authors:** Olivier Thériault, Mohamed Chahine

**Affiliations:** Department of Medicine, Centre de Recherche de l'Institut Universitaire en Santé Mentale de Québec, Université LavalQuebec City, QC, Canada

**Keywords:** voltage-gated sodium channel, neuronal excitability, pain, biophysical properties, dorsal root ganglia neurons

## Abstract

Voltage gated sodium channels (Na_v_ channels) play an important role in nociceptive transmission. They are intimately tied to the genesis and transmission of neuronal firing. Five different isoforms (Na_v_1.3, Na_v_1.6, Na_v_1.7, Na_v_1.8, and Na_v_1.9) have been linked to nociceptive responses. A change in the biophysical properties of these channels or in their expression levels occurs in different pathological pain states. However, the precise involvement of the isoforms in the genesis and transmission of nociceptive responses is unknown. The aim of the present study was to investigate the synergy between the different populations of Na_v_ channels that give individual neurons a unique electrophysical profile. We used the patch-clamp technique in the whole-cell configuration to record Na_v_ currents and action potentials from acutely dissociated small diameter DRG neurons (<30 μm) from adult rats. We also performed single cell qPCR on the same neurons. Our results revealed that there is a strong correlation between Na_v_ currents and mRNA transcripts in individual neurons. A cluster analysis showed that subgroups formed by Na_v_ channel transcripts by mRNA quantification have different biophysical properties. In addition, the firing frequency of the neurons was not affected by the relative populations of Na_v_ channel. The synergy between populations of Na_v_ channel in individual small diameter DRG neurons gives each neuron a unique electrophysiological profile. The Na_v_ channel remodeling that occurs in different pathological pain states may be responsible for the sensitization of the neurons.

## Introduction

Voltage gated sodium channels (Na_v_ channels) play an important role in nociceptive transmission and are intimately tied to the genesis and transmission of neuronal action potentials. Five isoforms (Na_v_1.3, Na_v_1.6, Na_v_1.7, Na_v_1.8, and Na_v_1.9) have been linked to nociceptive responses (Laird et al., 2002; Dib-Hajj et al., 2009; Xie et al., 2013; Garrison et al., 2014). The diversity of the biophysical properties and expression patterns of Na_v_ channels point to functional specialization (Hu et al., 2009). Moreover, previous studies emphasized the importance of different properties of Na_v_ in the neuronal excitability. For instance, the slow closed-state inactivation of Na_v_1.7 is thought to play an important role in the amplification of subthreshold depolarization and initiate AP firing in neurons (Blair and Bean, 2002; Cummins et al., 2007). The fast recuperation from the inactivated state of Na_v_1.8 is thought to sustain the relatively high frequency firing of small DRG neurons (Cummins and Waxman, 1997; Renganathan et al., 2001) while the current induced by the hyperpolarized activation of Na_v_1.9 could modulate the membrane voltage and alter the neuronal excitability (Herzog et al., 2001).

Furthermore, defect in biophysical properties of peripheral Na_v_ channels lead to diverse pain syndromes. Two inherited human neuropathic pain conditions, erythermalgia (Black et al., 2004) and paroxysmal extreme pain disorder (PEPD) (Fertleman et al., 2006), are associated with various missense (gain of function) mutations in SCN9A, the gene encoding the human Na_v_1.7 channel. PEPD is associated with defective fast inactivation, whereas erythermalgia is associated with a negative-shifted voltage-dependence of activation of Na_v_1.7 channels (Estacion et al., 2008). More recently, a remarkable congenital indifference to pain (CIP) (Ahmad et al., 2007) was described in consanguineous Pakistani and Canadian individuals, who are homozygous for a null mutation (complete loss of function) of Na_v_1.7 (Cox et al., 2006; Ahmad et al., 2007). It has also been reported that humans can harbor single nucleotide polymorphisms in SCN9A, which apparently confer different thresholds for pain susceptibility, depending on the functionality of the inherited alleles (Reimann et al., 2010). A variant of Na_v_1.8 has been reported in a family with painful peripheral neuropathy (Faber et al., 2012). Gain-of-functions mutations on Na_v_1.9 were reported to induce either loss of pain (Leipold et al., 2013) or episodic pain (Zhang et al., 2013).

The regulation of Na_v_ channels in sensory neurons is complex and may be a mechanism for conferring unique biophysical properties on the neurons (Chahine et al., 2005). Changes in the voltage-dependence, kinetics, and expression of Na_v_ channel subtypes in several models of nerve injury and inflammation used in pain research have been reported (Gold et al., 1998, 2003; Moore et al., 2002; Black et al., 2004; Gold and Flake, 2005; Thakor et al., 2009). These changes have been shown to induce hyperexcitability in primary afferent neurons (Moore et al., 2002; Song et al., 2003).

The aim of the study was to understand how the different Na_v_ channel α-subunits present in peripheral neurons interact to modulate neuronal excitability and induce the various electrophysiological profiles seen in these neurons. We hypothesized that individual Na_v_ paralogs contribute to their different biophysical properties to shape the action potential waveforms and overall excitability of peripheral neurons. Clarifying interactions between Na_v_ subtypes is an important step toward understanding the development of neuropathic pain, and the maturation and diversifications of neurons.

We used the patch-clamp technique in the whole-cell configuration to record Na_v_ currents and action potentials from acutely dissociated small diameter DRG neurons from adult rats (<30 μm). We also performed single cell qPCR on the same neurons. We compared Na_v_ channel transcripts in individual neurons and correlated them to the electrophysiological profiles of the neurons to assess their impact on excitability.

The ratio of the expression of mRNA reflected the proportion of Na_v_ channels at the membrane of the neurons. Moreover, the ratio of the different Na_v_ channel subtypes expressed in each neuron conferred a unique electrophysiological profile on the neuron. For instance, compared to the Na_v_1.8 and Na_v_1.9 channel subtypes, Na_v_1.7 channel displayed faster AP kinetics and shorter AP. Moreover, the presence of Na_v_1.7 decreased the threshold potential and accelerates the rise and the decay of the AP.

## Materials and methods

### DRG neuron dissection and culture

Neurons were isolated from L4-L5 DRG of adult male Sprague–Dawley rats weighting from 250 to 350 g (Wilmington, MA). Briefly, freshly removed ganglia were de-sheathed and enzymatically digested at 37°C for 20 min in DMEM containing 2 mg·ml^−1^ of type 4 collagenase (Worthington Biochemical Corp.). Trypsin (2.5 mg·ml^−1^, Sigma) was added, and the neurons were incubated for an additional 15 min. The ganglia were then dissociated mechanically by trituration using fire-polished Pasteur pipettes. The cell suspensions were centrifuged for 5 min at 1000 rpm at room temperature. The cell pellets were re-suspended in DMEM containing 4 mg·ml^−1^ of type 2S trypsin inhibitor (Sigma), layered on 7.5% BSA in Dulbecco's phosphate buffered saline (DPBS), and centrifuged at 1000 rpm for 5 min. After removing the supernatant, the pellet containing the neurons was re-suspended in DMEM containing 10% heat-inactivated horse serum and 5% FBS. The neurons were plated on poly-d-lysine-coated dishes and were kept in a 5% CO_2_ incubator at 37°C. The patch-clamp recordings were performed within 18 h. All experiments were performed according to the guidelines of the Canadian Council on Animal Care and were approved by the Université Laval Animal Care Committee.

### Whole-cell patch-clamp recording

Neurons ranging in diameter from 20 to 30 μm (mean short and long axis) measured using a graduated ocular were selected for recording. Whole-cell Na_v_ currents in DRG neurons were recorded using an Axopatch 200B (Molecular Devices) with the whole-cell configuration of the patch-clamp technique. pClamp v10 (Molecular Devices) was used for the pulse stimulations and recordings. Currents were filtered at 5 kHz, digitized at 100 kHz using a Digidata 1200 series AD converter (Molecular devices), and stored on a personal computer for later offline analysis. Series resistance was compensated 70–80%. When needed, linear leak current artifacts were removed by online leak subtraction. Capillaries used for electrodes were silanized prior to fashioning to reduce mRNA adsorption. The capillaries were soaked in a 5% (v/v) solution of dimethyldichlorosilane (Sigma) in chloroform for 20 min in a fume hood. They were then sterilized (15 min) and dried (15 min) in an autoclave (Lin et al., 2007). Fire-polished low-resistance electrodes (1 MΩ) were pulled from 8161 glass (Corning) and were coated with Sylgard (Dow-Corning) to minimize pipette capacitance.

AP recordings were obtained from DRG neurons using the whole-cell configuration of the patch-clamp technique. APs were generated by 5-ms, 50–300-pA rectangular current pulse injections followed by a 100-ms interpulse at the holding potential and then a 600-ms pulse. The sequence consisted of at least two recordings of evoked APs before and after the addition of 1 μM TTX.

For whole-cell voltage-clamp recordings, the intracellular pipette solution contained 10 mM NaCl, 140 mM CsF, 1 mM EGTA, and 10 mM HEPES. The pH was adjusted to pH 7.3 with 1 mM CsOH. The external solutions contained, the bath solution contained 35 mM NaCl, 105 mM choline chloride, 3 mM KCl, 1 mM CaCl_2_, 1.0 mM MgCl_2_, 10 mM glucose, 10 mM HEPES, and 100 nM CdCl_2_ to block calcium channels. The pH was adjusted to 7.3 with 1 M NaOH.

For the current-clamp recordings, the intracellular solution contained 122 mM KCl, 10 mM NaCl, 1.0 mM MgCl_2_, 1.0 mM EGTA, and 10 mM HEPES. The pH was adjusted to 7.3 with 1 M KOH. The extracellular solution contained 154 mM NaCl, 5.6 mM KCl, 2.0 mM CaCl_2_, 1.0 mM MgCl_2_, 10 mM glucose, and 8.0 mM HEPES. The pH was adjusted to 7.4 with 1 M NaOH.

### RT-qPCR

#### RNA extraction

We used two-step RT-qPCR to evaluate the mRNA in each DRG neuron tested. After the patch-clamp recording, the neuron was drawn into the recording pipette, expelled into a thin wall PCR tube, and stored in liquid nitrogen until the RT-PCR was performed. The RT-PCR reagents were added directly to the tube.

Total RNA for primer validation and standard curves was extracted from the DRG neurons using TRIZOL® (Invitrogen). RNA integrity was evaluated by ethidium bromide staining of 1% agarose gels. Total RNA was quantified by recording the optical density at 260 and 280 nm.

#### RT-PCR

RT-PCR was performed using Transcriptor First Strand cDNA Synthesis kits (Roche) according to the supplier's protocol. Briefly, lysed cells or 1 μg of total RNA was added to 60 pmol random hexamer primer and water was added to bring the volume to 13 μL. The template-primer mixture was denatured for 10 min at 65°C. Reaction buffer, RNase inhibitor (20 U final concentration), DNTP (1 mM each), and Transcriptor Reverse Transcriptase (1 U final concentration) were added to the mixture (20 μL). cDNA was synthesized at 50°C for 1 h. The enzymes were heat-deactivated at 85°C for 2 min. cDNA from DRG was stored at −80°C until used. Single cell qPCR was performed on the same day as the electrophysiological recordings.

#### qPCR

The qPCR assays were performed using a previously described protocol (Chatelier et al., 2012). Briefly, amplification products were detected with SYBR Green I and a LightCycler® 480 platform (Roche) using the supplier's protocol. Primers were designed using PerlPrimer v1.1.19 (Marshall, 2007). The qPCR samples were run at least in duplicate. A non-template control (NTC) and a positive control for each primer pair were included in each qPCR run. The qPCR conditions were as follows: an initial 7-min step at 95°C to activate the Taq polymerase and 45 cycles of 10 s at 95°C and 10 s at 58°C, followed by a 12-s elongation step at 72°C.

qPCR efficiency was determined using a series of known cDNAs, and the *Cq* values were plotted against the relative cDNA concentrations. qPCR efficiency was calculated using the slope of the regression line using the following equation: *E* = 10ˆ[−1/slope]. The analyses were performed using LightCycler® 480 SW 1.5 software. Quantifications were corrected for efficiency and run-to-run variations were adjusted using a known standard:

$$Normalized\;ratio={\left(\frac{\left[Target\right]}{\left[Reference\right]}\right)}_{sample}÷{\left(\frac{\left[Target\right]}{\left[Reference\right]}\right)}_{calibrator}$$

Target represents Cp of sodium channels and reference represents Cp of GAPDH (Gudrun, 2006). This is an efficient and accurate method to compensate for efficiency and run-to-run variations without the need of a standard curve in every run (Bustin, 2004). The specificity of the amplification for each run was monitored by melting curve analysis and was performed immediately following the PCR by continuously reading the fluorescence while slowly heating the reaction mixture from 65 to 95°C.

### Data analysis

Electrophysiological data were analyzed using a combination of pCLAMP software v10.0 (Molecular Devices), Microsoft Excel, and SigmaPlot 12.0 (Systat Software, Inc.).

Unless otherwise stated, statistical analyses were performed using SAS/STAT software v9.2 (SAS Institute Inc.). The results of Pearson analyses are expressed as coefficients of Pearson (ρ). Values are expressed as means ± s.e.m. When required for clarity, results are expressed as z-scores (z-scores = (x-mean)/SD).

Cluster analyses were performed to identify homogenous subsets of neurons based on the mRNA each subset expressed. mRNA quantifications were entered into the cluster analysis based on a Gaussian mixture model implemented in the R software with the Mclust package (available at http://cran.r-project.org/web/packages/mclust/index.html). Since we did not want to define a number of a priori subgroups of neurons, we compared the adjustments of different clustering solutions with 1, 2, 3, 4, and 5 clusters using the Bayesian Information Criterion (BIC). The inspection of the BIC values showed that the five-cluster solution worked best. However, given that there were very slight differences between two pairs of clusters, we opted for a three-cluster solution. Each subject was then assigned to a most probable cluster.

## Results

### qPCR amplification efficiency and single-cell qPCR validation

We validated the selectivity of the qPCR reaction using ethidium bromide-stained agarose gels. We observed single bands on the agarose gels, indicating that each primer pair produced one amplicon. The amplicons were validated by sequencing (data not shown). qPCR amplification was assessed using a melting curve analysis to ensure that it gave rise to a single PCR product. We evaluated the efficiency of the qPCR by generating standard curves over a wide range of mRNA copies using serial dilutions of known concentrations of cDNA (Supplement Figure 2A). Each primer pair had an efficiency superior to 1.90 in the 5-log range and was efficient with a low copy number (<100 copies) of mRNA (Table 1).

**Table 1:** **qPCR primers used**.

**Gene (protein)**	**Amplification length (pb)**	**Primer sequence**
*SCN3A* (Na_v_1.3)	158	F: 5′-AACGAAAGACGATCAAGACC-3′
		R: 5′-CCAAAGAAACATCAACGATCAG-3′
*SCN9A* (Na_v_1.7)	163	F: 5′-GGGAACTTGATCTTTACAGGG-3′
		R: 5′-ACTGATAATCCTTCCACATCTG-3′
*SCN10A* (Na_v_1.8)	189	F: 5′-TAGACATGGAGAAGAGGGAC-3′
		R: 5′-TTCAAGCTCCTCAATGACAG-3′
*SCN11A* (Na_v_1.9)	196	F: 5′-AAATGATCCTGAAGTGGGTG-3′
		R: 5′-GTAGACGACAACCTTCATTCC-3′
*GAPDH*	152	F: 5′-AGTATGTCGTGGAGTCTACTG-3′
		R: 5′-GGGAGTTGTCATATTTCTCGT-3′
*ACTB*	175	F: 5′-AGATCAAGATCATTGCTCCTCC-3′
		R: 5′-AACGCAGCTCAGTAACAGTC-3′
*PPIA*	160	F: 5′-TTTATCTGCACTGCCAAGAC-3′
		R: 5′-AATTAGAGTTGTCCACAGTCGG-3′

Since the quantification was relative to a reference gene, we verified the stability of the selected gene in individual cell experiments. We selected three references genes that are frequently used in qPCR experiments: GAPDH (Glyceraldehyde 3-phosphate dehydrogenase), ACTB (beta-actin), and PPIA (peptidylprolyl isomerase A). We first determined whether the ratio of the genes was stable in single neurons. Supplement Figures 1A,B show the cell-to-cell variations of the ACTB/GAPDH and PPIA/GAPDH gene ratios, respectively. The variations are expressed as (x-mean)/mean. We observed a marked cell-to-cell variation in the ACTB/GAPDH gene ratio of 59% with a mean error of 34%. The variation in the PPIA/GAPDH gene ratio was generally under 20% with a mean error of 12%. There was little correlation between the *Ct* values for ACTB and GAPDH, indicating that there was a large variation in the amount of mRNA among cells (Supplement Figure 1A). As such, GAPDH and ACTB could not be used as references genes. Supplement Figure 1D shows the correlation between the *Ct* values of GAPDH and PPIA for different cells. The high correlation (*R*^2^ = 0.98) and the slope value approaching one indicated that there was a low cell-to-cell variation for these two genes. Since GAPDH levels appeared to be stable in individual neurons, we used GAPDH as a reporter gene in our experiments.

We also ensured that the selectivity and efficiency were conserved in our experimental conditions by quantifying serial 1:1 dilutions (up to 1:32) of mRNA from single cells (Supplement Figure 2A). We observed very good quantification at all dilutions, with an error of less than10%. Every amplification product was monitored with a melting curve to ensure that we had a single amplification product.

RT-PCR is often inefficient and can be a source of error (Stahlberg et al., 2004). The relative quantification of the GAPDH reference gene minimizes the risk of error by reducing the influence of variation in the efficiency of the RT-PCR. We assumed that the efficiency was similar for each gene tested and, as such, that the ratios of the Na_v_ channel transcripts to the reference gene were similar, regardless the efficiency of the RT-PCR. The small amount of mRNA in a single cell and the small volume may also have induced an error. However, any loss of mRNA could be assumed to be the same for each gene we tested and, as such, would also be also minimized by the relative quantification.

### mRNAs reflect electrophysiological properties

**Figure 1:**
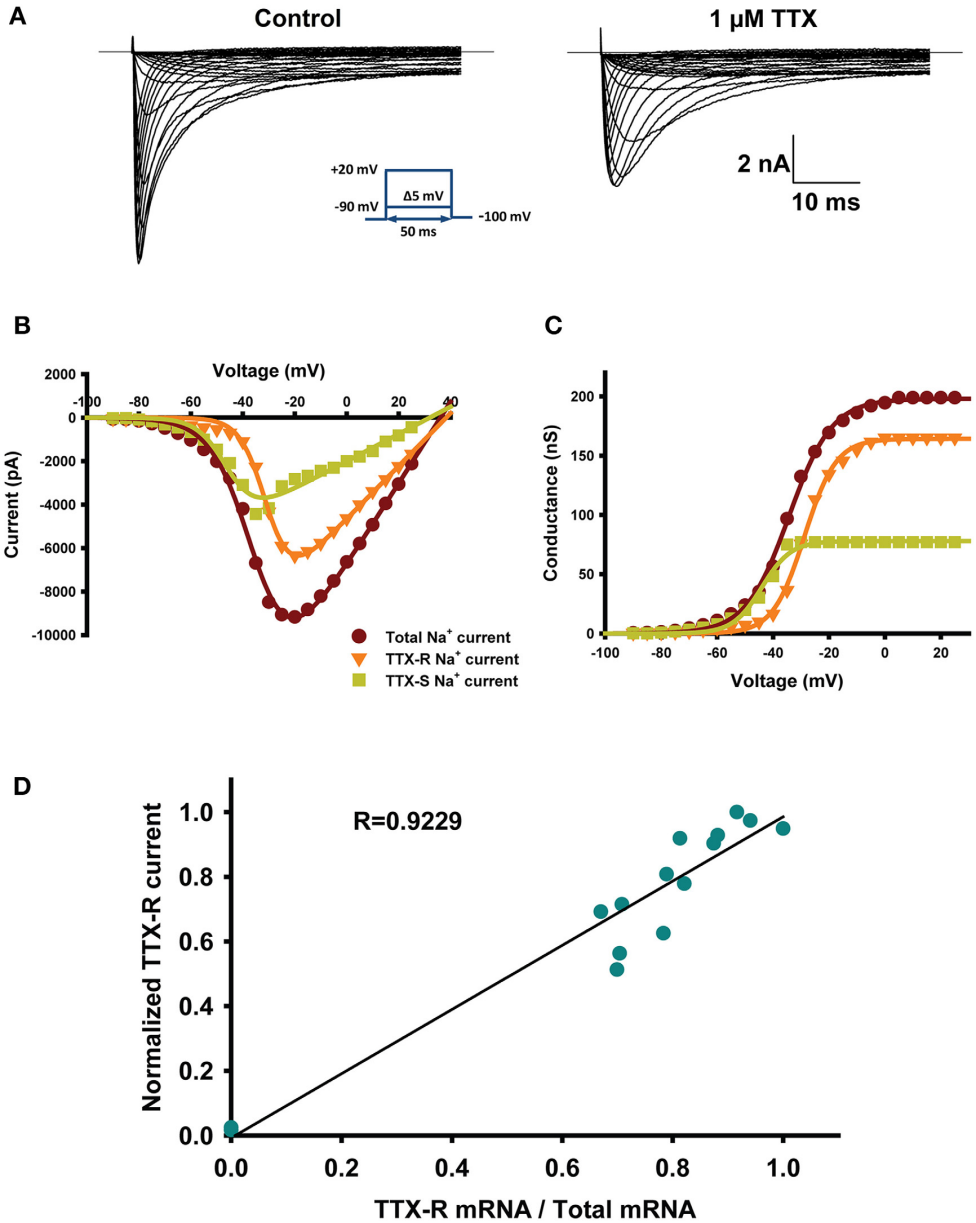
**mRNAs reflect electrophysiological properties. (A)** Representative current-traces recorded from small diameter DRG neurons (28 μm in diameter) before (right) and after (left) the addition of 1 μM TTX. The protocol is shown in the inset. **(B)** Example of current-voltage (I–V) relationships obtained from the small diameter DRG neuron shown in **(A)**. The dark red trace is the total current recorded under control conditions, the orange trace is the total TTX-R current recorded in presence of 1 μM TTX, and the green trace is the TTX-S current obtained by subtracting the TTX-R Na_v_ current (orange) from the total Na_v_ current (dark red). I–V are fitted using a Hodgkin–Huxley-like equation: *f* = *G*_max_*(*V* − *V*_rev_)/ (1 + exp((*V* − *V*_1/2_)/*k*)), where *G*_max_ is the maximal conductance, *V* the potential, *V*_1/2_ is the voltage at which half of the channels are in the open state, *V*_rev_ is the reversal potential, and k is the slope factor. **(C)** Activation curves obtained from **(B)** illustrating the conductance of total Na_v_ current (dark red), TTX-R current (orange) and TTX-S current (green). The conductance was calculated using the following equation: *G*_Na_ = *I*_Na_/(*V*_m_ − *V*_rev_), where *G*_Na_ is the conductance, *I*_Na_ is the peak current for the test potential *V*_m_, and *V*_rev_ is the reversal potential estimated from the current-voltage curve. **(D)** Correlation of normalized TTX-R Na_v_ currents to normalized mRNA coding for TTX-R channels (Na_v_1.8 + Na_v_1.9/(Na_v_1.7 + Na_v_1.8 + Na_v_1.9)). Currents were normalized to the maximal current. *n* = 17 from 8 animals.

The voltage-clamp approach can discriminate between TTX-sensitive and resistant Na_v_ channels. TTX-sensitive Na_v_ channels (TTX-S) are completely blocked by TTX at low nM concentrations while TTX-resistant Na_v_ channels (TTX-R) are insensitive to TTX (IC_50_ > 40 μM) (Caffrey et al., 1992). Figure 1A shows an example of a representative Na_v_ current recorded from a small neuron before (left) and after (right) the addition of 1 μM TTX. The TTX-R Na_v_ current was probably due to Na_v_1.8 and Na_v_1.9 channels. The TTX-S current-voltage relationship (I/V) curve was obtained by subtracting the I/V-curve of the TTX-R Na_v_ current from the total Na_v_ current (Figure 1B). Figure 1C shows the GV curves of TTX-R current and TTX-s current obtain from data of Figure 1B (Figure 1A). The proportions of the TTX-S and TTX-R Na_v_ currents were strongly correlated (*R*^2^ = 0.92) with the relative expression of mRNA by the TTX-S and TTX-R Na_v_ channels (Figure 1D). In these experiments Na_v_1.6 was not quantified due to low levels of transcripts in less than 35% of neurons (16 out of 46 neurons), data not shown. These results are similar to the study by Ho and O'leary where Na_v_1.6 is 6–8 time less abundant than Na_v_1.7, Na_v_1.8, and Na_v_1.9 (Ho and O'Leary, 2011).

### Impacts on AP properties

**Figure 2:**
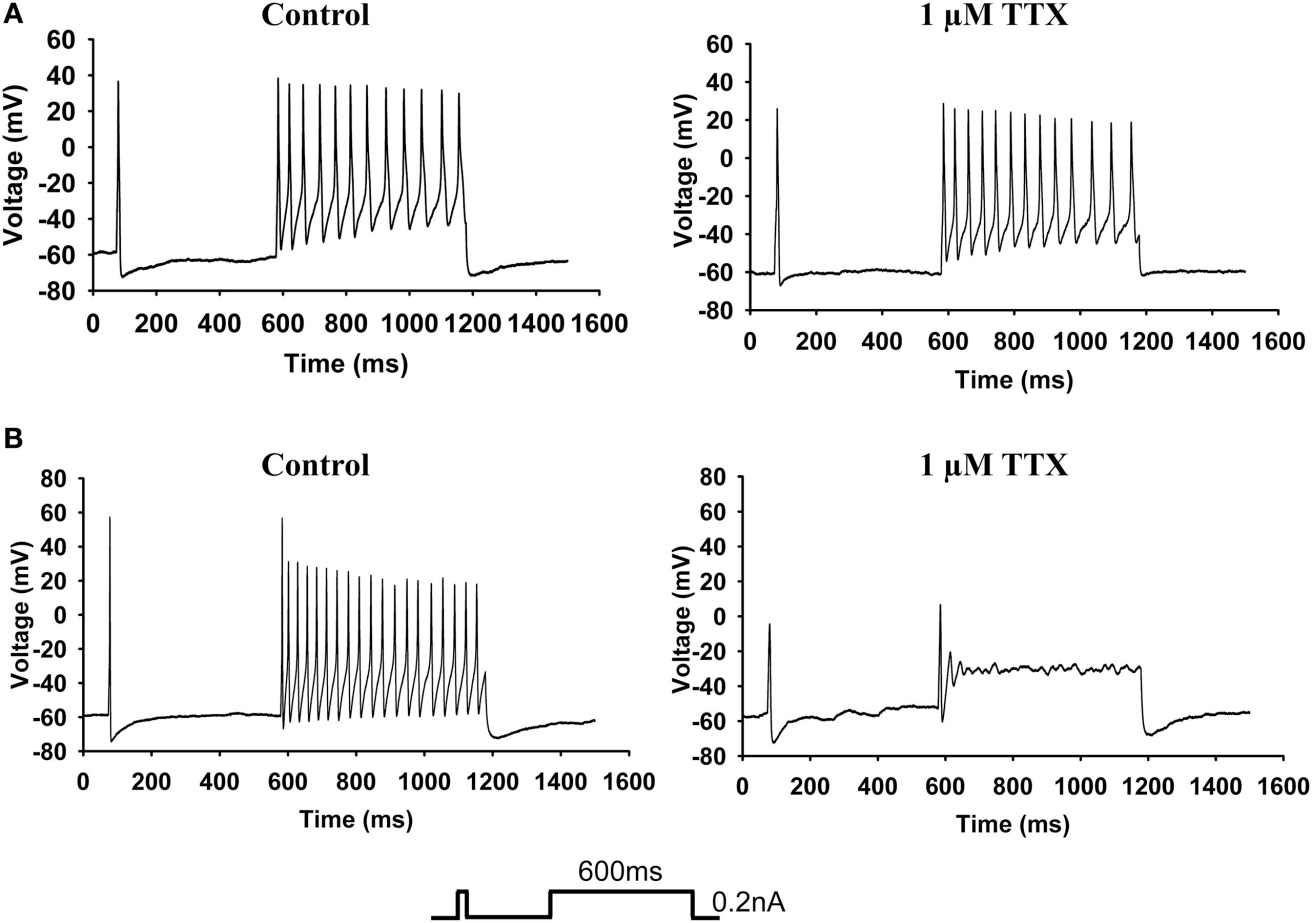
**Current-clamp analysis. (A)** Examples of AP firing (see protocol in inset) recorded from a 27-μm-diameter TTX-R neuron. The left panel shows the recording of AP firing before the addition of TTX, and the right panel shows the recording of AP firing by the same neuron after the addition of 1 μM TTX. **(B)** Examples of AP firing recorded from a 30-μm-diameter TTX-S neuron. The left panel shows the recording of AP firing before the addition of TTX, and the right panel show the recording of AP firing by the same neuron after the addition of 1 μM TTX.

We analyzed the parameters of APs recorded from small diameter DRG neurons in the current-clamp mode and quantified the mRNA in the neurons by single-cell qPCR. Neurons ranging in diameter from 20 to 30 μm exhibited marked differences in sensitivity to TTX. Twenty-one of the 49 neurons were sensitive to 1 μM TTX and exhibited no AP firing. Figure 2A shows a typical neuron in which AP firing was resistant to TTX. The left panel of Figure 2A show a representative AP recording under control conditions and the right panel shows a representative AP recording in the presence of 1 μM TTX. The protocol is shown in the inset. Figure 2B shows a neuron whose AP firing was inhibited by TTX. The left panel shows AP firing prior to the addition of TTX and the right panel show that the firing was abolished after the addition of 1 μM TTX. The first AP was not abolished by TTX.

Table 2 shows Pearson correlations between mRNAs and several biophysical properties of the AP. The correlations are expressed as negative or positive *r* values, which were considered significant at *p* < 0.05 (^*^p < 0.05, ^**^p < 0.01). There was a significant correlation between Na_v_1.7 mRNA and the overshoot, threshold (in mV and in pA), rise time (dV/dT), and time of decay as well as between Na_v_1.8 and Na_v_1.9 mRNA and the half AP width (duration of the AP at 50% amplitude), current threshold, and overshoot. There was also a significant correlation between Na_v_1.9 mRNA and a slowing of the rise and decay of dV/dT.

**Table 2:** **Pearson correlations of Na_v_ channels mRNA and electrophysiological properties measured**.

	**Frequency**	**Half AP width**	**Overshoot**	**Threshold (mV)**	**Threshold (pA)**	**dV/dT (decay)**	**dV/dT (rise)**
Na_v_1.7	–	−0.46^**^	0.43^**^	−0.32^*^	−0.46^**^	−0.30^*^	0.46^**^
Na_v_1.8	–	0.31^*^	−0.31^*^	–	0.45^**^	–	–
Na_v_1.9	–	0.34^*^	0.38^**^	–	0.32^*^	0.29^*^	−0.37^**^

Data are presented as the correlation coefficient of Pearson correlations (ρ). Significant values are presented with ^*^;

* p-value < 0.05;

** *p-value < 0.01; –, no significant correlation*.

### Cluster analysis

**Figure 3:**
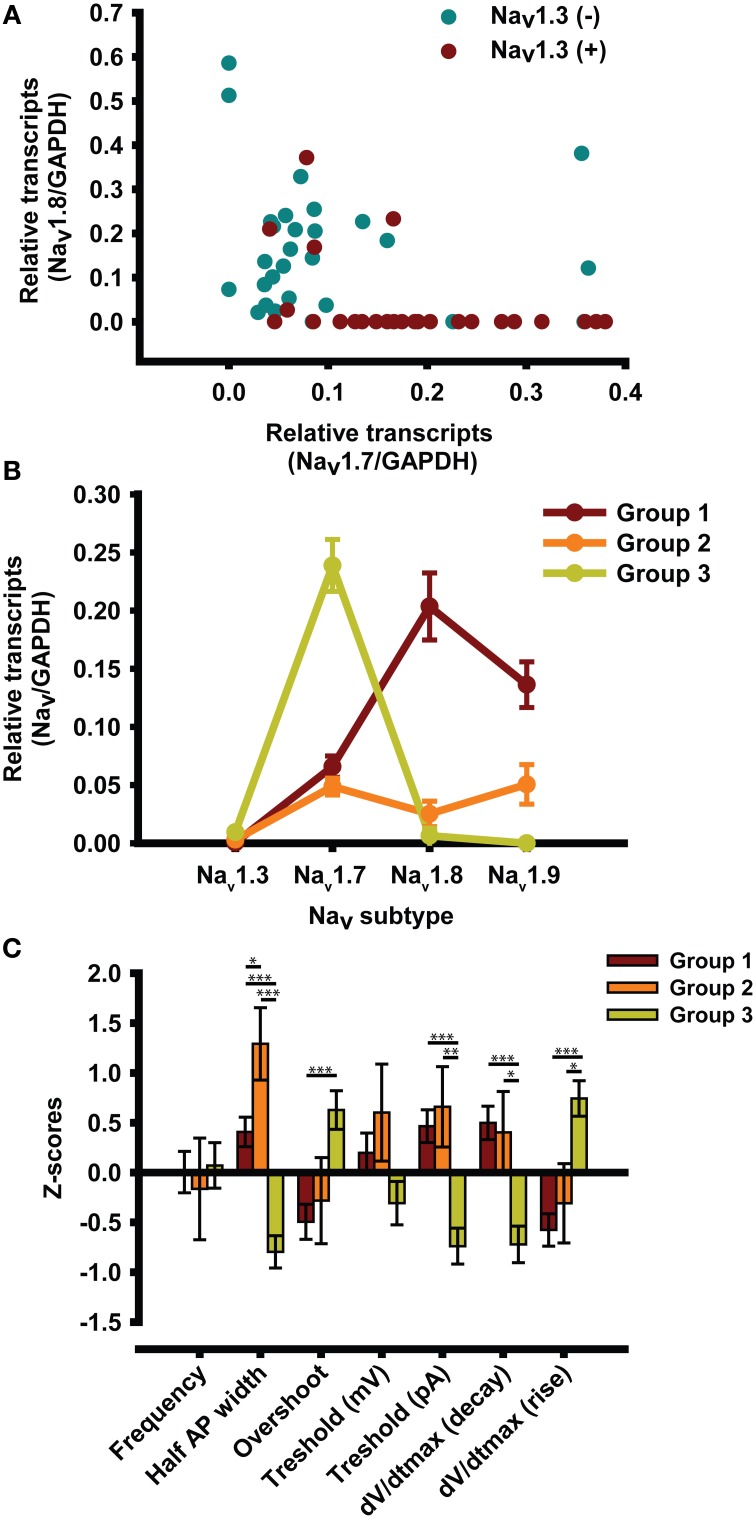
**Cluster analysis. (A)** Graphical representation of the distribution of Na_v_ mRNA as a function of the presence of Na_v_1.3 mRNA. **(B)** Cluster analyses of mRNA from single DRG neurons revealed three profiles that represent 49, 8, and 43% of all the small diameter DRG neurons tested (<30 μm). **(C)** Electrophysiological characterization of the three profiles expressed as z-scores ± s.e.m. Neurons are recorded without TTX. z-scores = (x-mean)/*SD*; ^*^p < 0.05; ^**^p < 0.01; ^***^p < 0.001, *n* = 49 from 16 animals.

We performed another *post-hoc* analysis of the data by plotting the amounts of mRNA in order to visualize their distributions (Figure 3A). Na_v_1.7 mRNA was plotted against Na_v_1.8 mRNA in Na_v_1.3 mRNA-positive (red) and Na_v_1.3 mRNA-negative cells (blue). We observed a marked difference between the two types of cell, with Na_v_1.3 mRNA-positive cells expressing more Na_v_1.7 mRNA than Na_v_1.3 mRNA-negative cells.

We also performed a cluster analysis to determine whether there were different subgroups of neurons (Figure 3B). Interestingly, the cluster analysis revealed that there were three subgroups of neurons that differed in the expression of Na_v_ channel mRNA. The first subgroup (red) expressed large numbers of TTX-R Na_v_1.8 and Na_v_1.9 channels. The second subgroup (orange) made up 8% of all the neurons tested and expressed a combination of TTX-S Na_v_ channels (Na_v_1.7) and TTX-R Na_v_ channels (Na_v_1.8 and Na_v_1.9). The third subgroup (green) made up 43% of all the neurons tested and mainly expressed TTX-S Na_v_1.7 Na_v_ channels.

**Figure 4:**
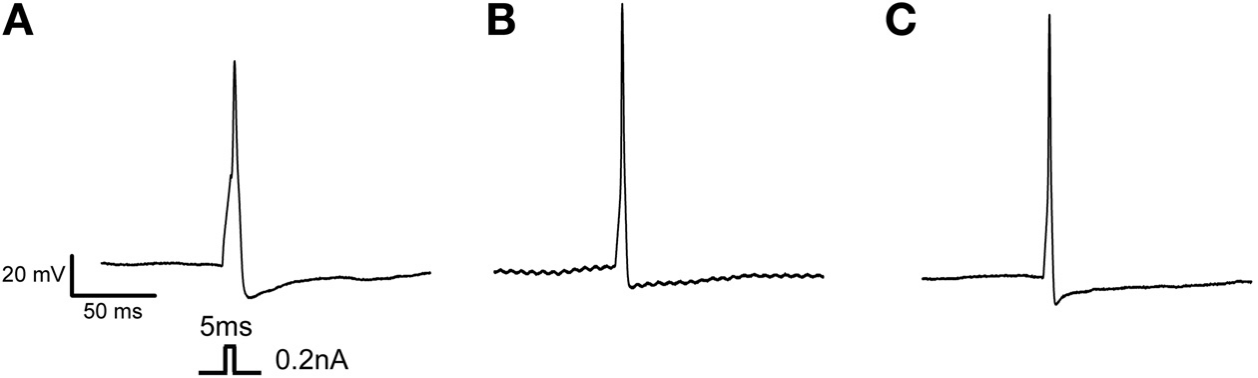
**Action potential properties of distinct groups. (A)** Example of single AP firing elicited from a 5 ms pulse recorded from a neuron of the first group expressing principally TTX-R Na_v_ channel. **(B)** Example of single AP firing elicited from a 5 ms pulse recorded from a neuron of the second group, expressing a combination of TTX-S and TTX-R Na_v_ channel. **(C)** Example of single AP firing elicited from a 5 ms pulse recorded from a neuron of the third group, expressing principally TTX-S Na_v_ channel. Protocol is shown in inset.

We performed statistical analyses to determine whether there were any differences in AP parameters between the subgroups. Results are expressed as z-scores on the y axis (Figure 3C). We also performed multiple comparisons when the ANOVA *p*-value was less than 0.05 (*p* < 0.05). We observed significant differences between subgroups 1 and 3 (^*^) and subgroups 2 and 3 (¤). There was a significant difference between subgroups 1 and 2 vs. subgroup 3 in terms of half AP width, overshoot, current threshold, and maximum dV/dt rise. There was also a significant difference between groups 1 and 3 in terms of dV/dt decay. The Figure 4 illustrates the AP properties of a representative neurons from each groups.

## Discussion

One concern with qPCR is that the amount of amplified mRNA is not proportional to the amount of functional protein (Greenbaum et al., 2003; Maier et al., 2009). We recorded voltage-clamp currents and performed single-cell qPCR to measure the expression of functional Na_v_ channels and mRNA (Sucher et al., 2000; Lin et al., 2007). Adding TTX makes it possible to discriminate between TTX-R and TTX-S Na_v_ channels and, as such, correlate the expression of functional TTX-R and TTX-S Na_v_ channels and their mRNAs. We observed a strong correlation between the proportion of mRNA and the expression of functional Na_v_ channels (Figure 1D), indicating that the expression of mRNA was a good representation of the amount of functional protein in our study and could thus be used to quantify the amount of proteins.

There was a significant correlation between Na_v_ channel mRNA and AP parameters. It has been shown that faster Na_v_ channel kinetics may result in faster AP kinetics (Cummins et al., 2007; Schild and Kunze, 2012). The presence of Na_v_1.7 channels increased the rise in dV/dT, decreased the decay in dV/dT, and reduced the half AP width of the AP. The correlation analysis also showed that Na_v_1.7 channels had a lower threshold and a higher overshoot than Na_v_1.8 and Na_v_1.9 channels while Na_v_1.8 and Na_v_1.9 channels displayed slower AP kinetics than Na_v_1.7 channels. This is in agreement with the hypothesis that Na_v_1.7 amplifies subthreshold stimuli to evoked AP (Blair and Bean, 2002). Moreover, those results can be linked to studies that indicate a role for the upregulation of Na_v_1.7 in neuropathic pain (Black et al., 2004; Cummins et al., 2004).

In our study, the presence of Na_v_1.9 and Na_v_1.8 channels significantly slowed dV/dT rise and decay (*p* = 0.06 for Na_v_1.9 and *p* = 0.05 for Na_v_1.8). The half AP width was also correlated with the proportion of Na_v_1.8 and Na_v_1.9 Na_v_ channels, which is in agreement with their slower kinetics (Vijayaragavan et al., 2001). Those results are in accordance with studies which concluded that the sodium channels subtypes have different roles in the electrogenesis within DRG neurons (Rush et al., 2007; Ho and O'Leary, 2011).

Pearson analysis does not show any correlation between Na_v_ channels transcripts and firing frequency of the neurons (Table 2). It has been assumed that slowly inactivating Na_v_1.8 channels support high frequency firing in small C-fiber neurons (Renganathan et al., 2001). However, our data showed that both Na_v_1.7 and Na_v_1.8 channels can support high frequency tonic firing in DRG neurons. Indeed, Na_v_1.7 supports high frequency neurons in SCG neurons (Rush et al., 2006).

The plot of the distributions of Na_v_1.7 and Na_v_1.8 mRNA showed marked differences depending on whether Na_v_1.3 mRNA was present or not, which together with prior observations indicated that there are several subtypes of neurons (Fornaro et al., 2008; Ho and O'Leary, 2011). We thus performed a cluster analysis of mRNA distributions, which revealed that there are three subgroups of neurons in small diameter DRG neurons (<30 μm) that we tested. One subgroup mainly expressed Na_v_1.8 and Na_v_1.9 mRNA, the second subgroup expressed similar amounts of Na_v_1.7, Na_v_1.8, and Na_v_1.9 mRNA, while the third subgroup mainly expressed Na_v_1.7 mRNA.

The three subgroups displayed significant differences in AP properties. The frequency and threshold (in mV) were the only parameters that were the same for all three subgroups. The half AP width, overshoot, current threshold, dV/dT decay, and dV/dT rise of the third group differed from those of the first and the second subgroups. The lack of significant differences between the first and second subgroups may be due to the very low proportions of these two subgroup and greater differences between them.

It is interesting that the biophysical properties of the three subgroups were similar to the results of the Pearson correlations. The third subgroup, which had a greater proportion of Na_v_1.7, had a faster AP, a shorter half AP width, and a faster dV/dT decay and rise than the first and second subgroups. The third subgroup also had a greater overshoot and a lower current threshold than the first and second subgroups. The first and second subgroups, which had more Na_v_1.8 and Na_v_1.9 channels than Na_v_1.7 channels, exhibited slower kinetics than the third subgroup as well as a lower overshoot and a higher current threshold.

Potassium and calcium channels also have a major impact on the firing pattern (Deister et al., 2009; Winlove and Roberts, 2012). It is thus possible that the changes we observed were caused by more than one contributing factor (ion channels, pumps, exchangers, etc.). Nevertheless, the strong linear correlation indicated that Na_v_ channels play a major role in the biophysical properties of DRG neurons. It's important to keep in mind that electrophysiological properties of a neuron are the results of the equilibrium between the different ion channels present and therefore changes might not lead to the same outcome. As shown by Rush et al., a gain of function mutation of Na_v_1.7 that lead to erythermalgia render the sensory neurons hyperexcitable and sympathetic neurons hypoexcitable (Rush et al., 2006).

Our results also have major implications during development since the different expression patterns that occur during the maturation of the nervous system (Beckh, 1990) are associated with changes in the electrical excitability of the neurons (Benn et al., 2001). Our results contribute to understanding how the different subtypes of Na_v_ channels affect the maturation and excitability of sensory neurons.

While there are numerous reports in the literature on the modulation of Na_v_ channels and the changes in the excitability of sensory neurons during different pathological conditions, little is known about how this affects the excitability of neurons (Cummins and Waxman, 1997; Berta et al., 2008; Thakor et al., 2009). We showed that the modulation of Na_v_ channels has an impact on the firing properties of acutely dissociated DRG sensory neurons. It now remains to be determined how changes in Na_v_ channel expression in pathological conditions affect the excitability of neurons.

We concluded that the different Na_v_ channel subtypes in small diameter DRG neurons point to complex physiological interactions and that their modulation affects the biophysical properties of these neurons. Further studies are needed to unravel the roles of the Na_v_ channel subtypes during development and in pathological conditions.

### Conflict of interest statement

The authors declare that the research was conducted in the absence of any commercial or financial relationships that could be construed as a potential conflict of interest.
