# Supplementary material for: Correlation of the electrophysiological profiles and sodium channel transcripts of individual rat dorsal root ganglia neurons
Source: Front Cell Neurosci. 2014 Sep 19;8:285. doi: 10.3389/fncel.2014.00285 (PMC4168718; doi:10.3389/fncel.2014.00285)
Supplement: Supplementary file 1 [file Image1.PDF]

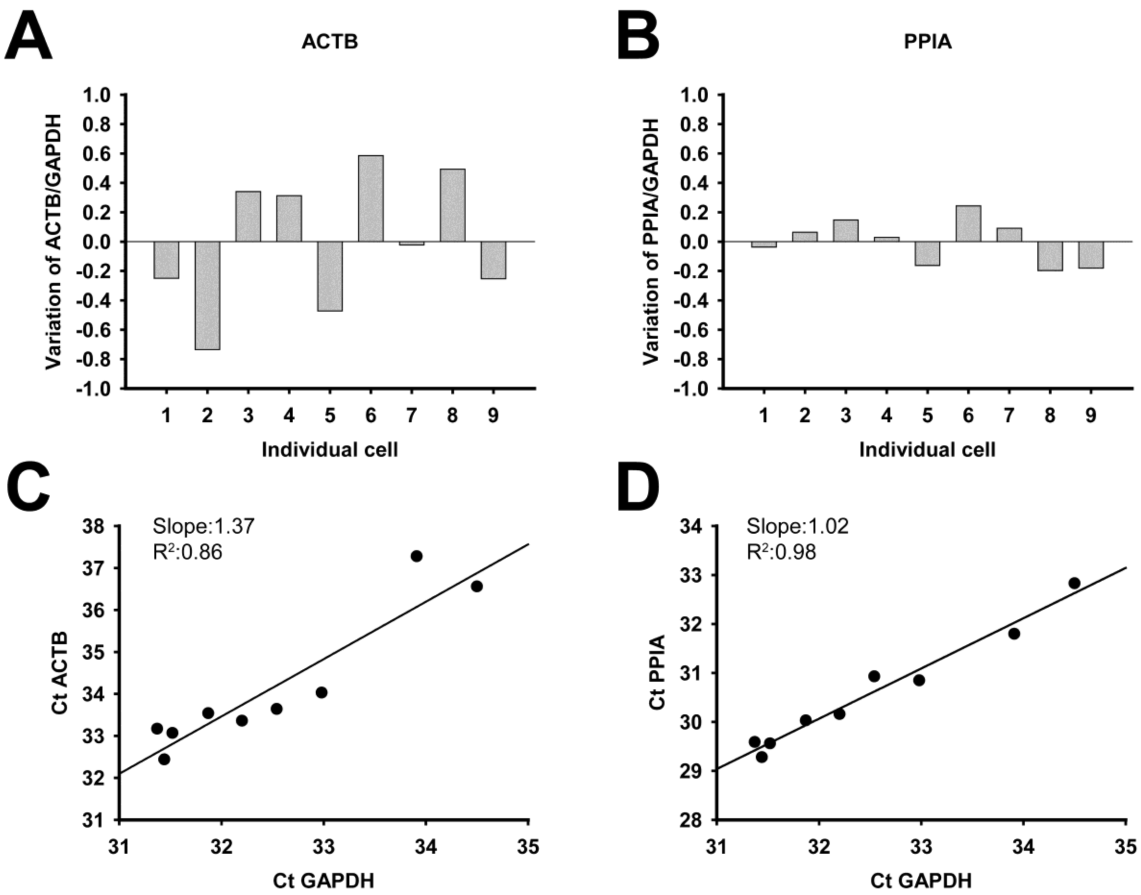

Supplement figure 1. Validation of reference genes
(A) Cell-to-cell variations in the C_t value of the ACTB/GAPDH ratio. The Y axis represents the variation: (x-mean)/mean. **(B)** Cell-to-cell variations in C_t value of the PPIA/GAPDH ratio. The Y axis represents the variation: (x-mean)/mean. (**C)** Conservation of the quantification when the C_t value changes. The X axis represents the C_t value of GAPDH in a single cell, and the Y axis represents the C_t value of ACTB in the same cell. **(D)** Conservation of the quantification when the C_t value changes. The X axis represents the C_t value of GAPDH in a single cell and the Y axis represents the C_t value of PPIA in the same cell. (n=9)
