# Supplementary material for: Correlation of the electrophysiological profiles and sodium channel transcripts of individual rat dorsal root ganglia neurons
Source: Front Cell Neurosci. 2014 Sep 19;8:285. doi: 10.3389/fncel.2014.00285 (PMC4168718; doi:10.3389/fncel.2014.00285)
Supplement: Supplementary file 2 [file Image2.PDF]

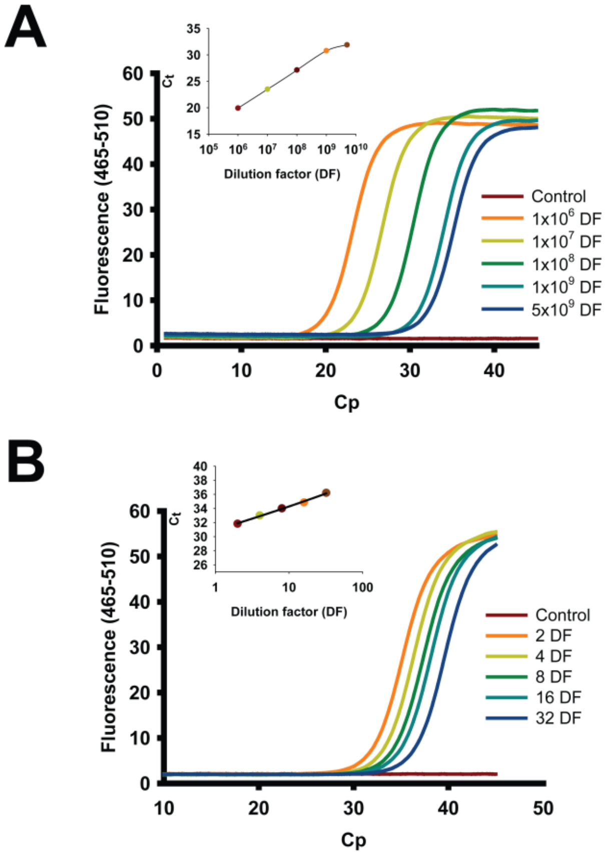

Supplement figure 2. qPCR validation and quantification

(A) Example of an amplification curve of serial 1:9 dilutions of GAPDH used to calculate the efficiency. The inset is the linear plot of the Cp values calculated from the dilutions and shows that the efficiency is conserved for low copy numbers (efficiency 1.90) Efficiency is calculated using the following function: $E = 10^{[1/slope]}$; when C_t is plotted against log(dilution). DF: Dilution factor. **(B)** Example of an amplification plot of serial 1:1 dilutions of GAPDH from a single cell RT-qPCR. The inset is the linear plot of the Cp values calculated from the serial dilutions of a single cell and shows that the efficiency is conserved up to a 1:32 dilution (efficiency 1.98).
